# Effect of Warming on Soil Fungal Community Along Altitude Gradients in a Subalpine Meadow

**DOI:** 10.3390/microorganisms12122527

**Published:** 2024-12-07

**Authors:** Jing Yin, Dandan Yuan, Jing Lu, He Li, Shuzheng Luo, Jianhua Zhang, Xingjia Xiang

**Affiliations:** 1 School of Resources and Environmental Engineering, Anhui University, Hefei 230601, China; yinjing01102023@163.com (J.Y.); yuandandan415@163.com (D.Y.); lujing22163@163.com (J.L.); 2 Anhui Province Key Laboratory of Wetland Ecosystem Protection and Restoration, Hefei 230601, China; 3 School of Geography, Geomatics and Planning, Jiangsu Normal University, Xuzhou 221116, China; lihe@jsnu.edu.cn; 4 Department of Biology, Xinzhou Normal University, Xinzhou 034000, China; luoshuzhengyan@163.com

**Keywords:** subalpine meadow, warming, altitude gradients, fungal community

## Abstract

The subalpine grassland ecosystem is sensitive to climatic changes. Previous studies investigated the effects of warming on grassland ecosystems at a single altitude, with little information about the response of subalpine meadows to warming along altitude gradients. This study aimed to evaluate the effects of warming on aboveground grass, belowground soil properties, and fungal community along altitude gradients in the subalpine meadow of Mount Wutai using the high-throughput sequencing method. Warming reduced the restriction of low temperatures on the growth of subalpine grass, resulting in increasing grass biomass, community height, and coverage. More grass biomass led to higher soil organic carbon resources, which primarily affected fungal community composition following warming. Warming might induce more stochastic processes of fungal community assembly, increasing fungal diversity at low altitudes. In contrast, warming triggered more deterministic processes to decrease fungal diversity at medium and high altitudes. Warming might improve the efficiency of soil nutrient cycling and organic matter turnover by increasing the relative abundance of soil saprotrophs and improving fungal network connectivity. The relative abundance of certain grass pathogens significantly increased following warming, thereby posing potential risks to the sustainability and stability of subalpine meadow ecosystems. Overall, this study comprehensively evaluated the response of the subalpine meadow ecosystems to warming along altitude gradients, clarifying that warming changes soil fungal community composition at different altitudes. The long-term monitoring of pathogen-related shifts should be conducted in subalpine meadow ecosystem following warming. This study provided significant scientific insights into the impact of future climatic changes on subalpine ecosystems.

## 1. Introduction

The Intergovernmental Panel on Climate Change (IPCC) has predicted that the global average temperature will continue to increase by 2.0–4.9 °C by the end of the 21st century [1]. The global climatic change may exceed the carrying capacity threshold of Earth’s ecosystems [2,3], implying that many ecosystems will face severe risks and irreversible impacts [4], seriously threatening the sustainable development of human society and natural ecosystems [5,6]. Soil contains the most diverse microbial communities on Earth, playing a crucial role in the biochemical processes of terrestrial ecosystems, such as organic matter decomposition and nutrient cycling [7,8]. The belowground microbial communities serve as the center of interaction between plants and soil. The increase in temperature affects ecosystem functions by altering the metabolic activity of soil microorganisms and the relationship between aboveground plants and belowground microorganisms [9,10]. The soil fungal community, as an important microbial indicator for measuring soil ecological status, can not only reflect the quality of terrestrial ecosystems but also provide rapid responses to environmental stress [11,12]. Therefore, understanding the ecological functions of soil fungal communities and their response to climate warming is of pioneering significance.

As a type of terrestrial ecosystem, a subalpine meadow not only plays critical ecological roles in regulating climate, maintaining soil moisture, and preserving biodiversity but also provides food sources for livestock, contributing significantly to the economy [13,14]. Additionally, the subalpine grassland ecosystem is extremely sensitive to climatic changes [15], making it an ideal experimental site for monitoring climatic changes and a key area for biodiversity conservation research. Progress has been made in studying the response of microbial communities to climate warming based on experiments conducted at alpine meadow ecosystems [16,17,18]. However, warming experiments along altitude gradients to comprehensively evaluate the impact of warming on a subalpine meadow ecosystem are less abundant.

The subalpine meadow system of Mount Wutai provides an opportunity to examine the impact of warming at different altitudes. Mount Wutai is located in Xinzhou City, Shanxi Province, China. The subalpine meadow of Mount Wutai is the highest-altitude, largest, and most typical subalpine summer pasture in North China, covering an area of 1069 km^2^. In recent years, the subalpine meadow of Mount Wutai has suffered substantial degradation, with a reduction in ecosystem services and production functions. This degradation is attributed to the combined effects of climate change, overgrazing, tourism, and various human activities, severely threatening regional ecological security [19].

The primary objective of this study was to explore the responses of the aboveground grass community, belowground soil properties, and fungal community to experimental warming in alpine meadows along altitude gradients of Mount Wutai. The findings of this study may provide experimental evidence and a theoretical basis for maintaining the stability of subalpine meadow ecosystems in the face of global warming. We hypothesized that: (1) warming promotes plant growth, with more organic carbon entering the soil through root exudates and litter, leading to an increase in soil organic carbon. (2) Warming alters soil fungal diversity, fungal community composition, and potential functions.

## 2. Materials and Methods

### 2.1. Study Site

The study area, the subalpine meadow of Mount Wutai (38°27′–39°15′ N, 112°48′–113°55′ E), is located in the northeast of Xinzhou City, Shanxi Province, China. Mount Wutai ranges in altitude from 610 to 3061 masl and has a temperate continental monsoon climate. An altitude of 2600–3000 masl was selected as the warming experimental area for this study. This area had typical periglacial landforms. The average annual precipitation was 668 mm [20]. The dominant species in the subalpine meadows of Mount Wutai included mainly *Kobresia pygmaea*, *Carex* sp., and *Viola philippica*.

### 2.2. Experimental Design

Hexagonal open-top chambers (OTCs) were selected for warming experiments [21]. The chamber design was modified from the Tibetan Plateau *Kobresia pygmaea* meadow experiment [22], with an upper side length of 40 cm, a lower side length of 75 cm, and a vertical height of 60 cm. The chamber frame was constructed of triangular steel, with transparent tempered glass fixed on the sides, providing more than 95% glass transmittance (Figure S1). The warming experiments began in August 2020 across four altitude gradients: high altitude (3000 masl), medium-high altitude (2900 masl), medium altitude (2800 masl), and low altitude (2600 masl). Each treatment consisted of five replicates. The paired control grassland plots of the same size were positioned 2 m away from each warming plot. Fences were installed in the experimental area to prevent animal damage. During the study period, air temperature increased by 1.7–3.4 °C, and the soil temperature increased by 1.6–3.3 °C, compared with the control (Table S1).

### 2.3. Sample Collection

Soil samples were collected using soil drills along diagonal lines at a depth of 0–10 cm from each plot. Upon collection, the samples were immediately transported to the laboratory to remove plant roots and stones and then passed through a 2 mm steel sieve. The samples were divided into two groups: one group of samples was stored at 4 °C for biochemical analysis, whereas another group of samples was stored at −20 °C for DNA extraction.

### 2.4. Analysis of Grass and Biochemical Properties

The grass survey was conducted on 15 August 2023, during the peak of the grass growth season, to obtain grass data. The dominant grass species are listed in Table S2. The grass height was measured using a steel tape measure. The coverage was assessed using the grid method. The aboveground grass biomass was harvested using the cutting method and then dried at 75 °C for 48 h until reaching a constant weight [23].

The soil pH was determined using a pH meter (ZD-18, Taizhou, Jiangsu, China), and soil moisture (SM) was determined using the oven-drying method (at 105 °C for 24 h until reaching a constant weight) [24]. The soil organic carbon (SOC) content was determined using the potassium dichromate oxidation method, and the soil dissolved organic carbon (DOC) content was determined using the fumigation extraction method [25]. The total nitrogen (TN) and total phosphorus (TP) contents were determined using the Kjeldahl digestion–distillation method and molybdenum blue colorimetry method, respectively [26,27]. Available phosphorus (AP) was extracted using sodium bicarbonate [28], and soil-dissolved organic nitrogen (DON) content was determined using the subtraction method (DON = TDN − DIN) [29]. The ammonium nitrogen (NH_4_^+^-N) and nitrate nitrogen (NO_3_^−^-N) contents were determined using a UV-visible spectrophotometer (Shimadzu, Kyoto, Japan) [30].

### 2.5. Soil DNA Extraction and Illumina MiSeq Sequencing

A total of 42 soil samples were collected, including 5 warming samples and 5 control samples at 2600 masl, 2800 masl, 2900 masl, respectively, and 6 warming samples and 6 control samples at 3000 masl. Soil DNA was extracted using FastDNA^®^ SPIN Kit (MP Biomedicals, Irvine, CA, USA). The extracted DNA was dissolved in 60 μL of TE buffer, quantified using NanoDrop ND-1000 spectrophotometer (Thermo Fisher Scientific, Waltham, MA, USA), and stored at −20 °C. The fungal ITS region was amplified using primers ITS1-F/ITS2-R [31]. PCR was carried out in 50 μL reaction mixtures containing the following: 25 µL of DNA polymerase (TaKaRa, Shiga, Japan); 1 µL of DNA template, 0.5 µL of forward primer, 0.5 µL of reverse primer, and 23 µL of double distilled water (ddH_2_O). The PCR conditions included 35 cycles (95 °C for 45 s, 56 °C for 45 s, and 72 °C for 45 s) with a final extension at 72 °C for 10 min. Then, all PCR products were quantified using an agarose gel DNA purification kit (TaKaRa, Japan). High-throughput sequencing analysis of fungal rRNA genes was performed using the Illumina MiSeq platform (Biozeron Biotechnology, Shanghai, China).

### 2.6. Processing of Sequencing Data

Raw fungal data were processed using the Quantitative Insights Into Microbial Ecology software (QIIME2-2021.2) [32], employing the “deblur” algorithm to filter out low-quality sequences. High-quality sequences were clustered into amplicon sequence variants (ASVs) with 100% similarity. Chimeric sequences were removed using the “vsearch” method, and the annotation was conducted using the classify-sklearn method [33] with the UNITE (7.2) database [34]. Subsets of 28,174 sequences per sample were randomly selected for further analyses.

### 2.7. Statistical Analysis

Paired-samples *t*-test analysis was conducted to evaluate the differences in grass diversity, soil properties, and fungal data between control and warming samples. One-way analysis of variance was performed to examine the differences in grass diversity, soil properties, and fungal data along altitude gradients. The differences in fungal community composition were analyzed using nonmetric multidimensional scaling (NMDS) and permutational MANOVA (ADONIS; permutations = 999) using the vegan package (v.2.6-4) in R-software (v.4.2.1). The Spearman analysis was used in Statistical Package for the Social Sciences (SPSS v.20.0) to test the significance of correlation coefficients. The indicator analysis was conducted using the labdsv package (v.2.1-0) in R-software (v.4.2.1). The Mantel test was performed using the vegan package (v.2.6-4) in R-software (v.4.2.1) to identify biochemical factors and grass biomass correlated with community composition. The fungal functions were predicted using the FUNGuild (v.1.1) analysis. The abundance null deviation value (NDV) was calculated using the vegan package (v.2.6-4) in R-software (v.4.2.1). The co-occurrence network analysis was performed using R-software (v.4.2.1) and Gephi v.0.9.2 software [35].

## 3. Results

### 3.1. Soil and Grass Properties

The warming showed minimal effect on soil pH (Figure S2A). The SM significantly decreased following warming, except at low altitude (Figure S2B). The SOC, DOC, and soil TN contents significantly increased following warming compared with the control (Figure S2C–E). Warming decreased DON content, except at low altitude, and decreased soil AP content (Figure S2F,G). Warming showed little effect on soil TP, NH_4_^+^-N, and NO_3_^−^-N contents compared with the control (Figure S2H–J). The effect of altitude on soil properties was shown in Figure S3. The soil pH and SM exhibited little difference along altitude gradients in both control and warmed soils (Figure S3A,B). The SOC and TN contents were lower in low-altitude soil under control treatment (Figure S3C,E).

**Figure 1:**
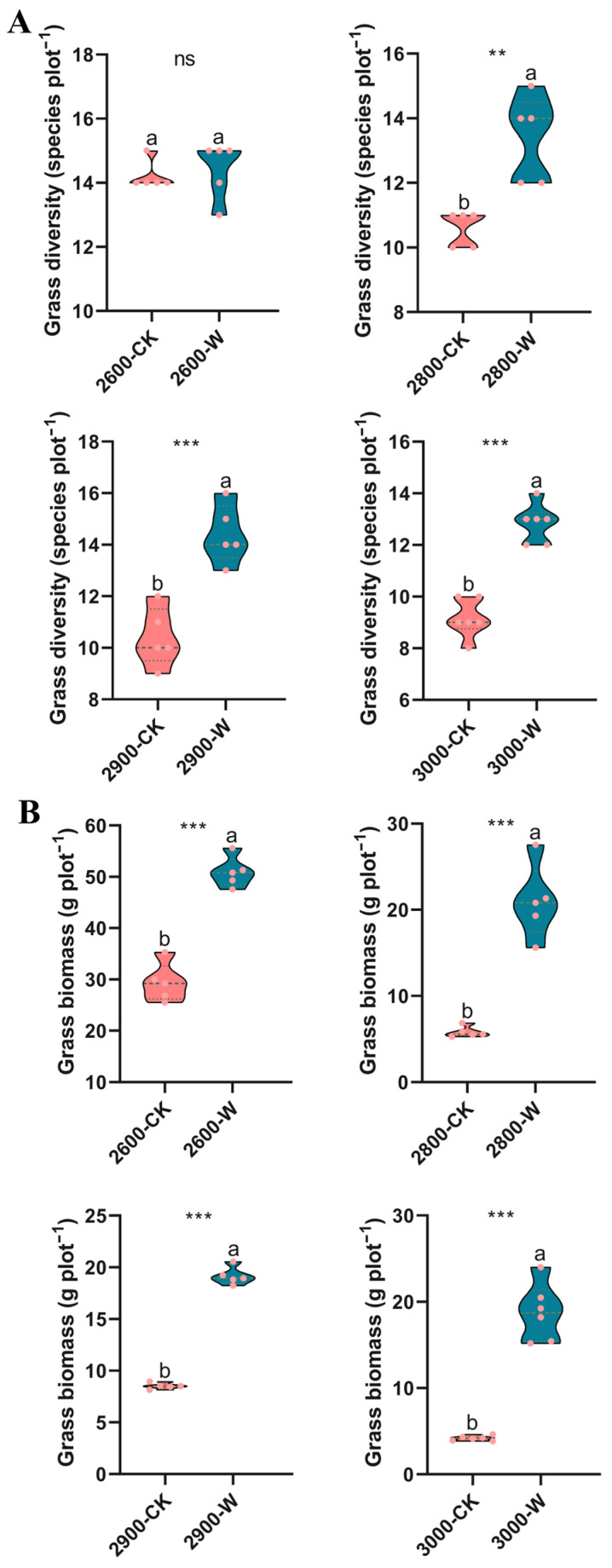
Effects of warming on aboveground grass diversity (**A**) and biomass (**B**) along altitude gradients. Letters represent significant differences from paired-samples *t*-test analysis. CK: control; W: warming. ns: *p* > 0.05; **: *p* < 0.01; ***: *p* < 0.001. Low altitude (2600 masl), medium altitude (2800 masl), medium-high altitude (2900 masl), and high altitude (3000 masl).

Warming increased grass diversity, except at low altitude, compared with the control (Figure 1A). The grass diversity showed a decreasing trend along altitude gradients, but with little difference following warming (Figure S4A). The grass biomass significantly increased at all four altitudes following warming (Figure 1B). The grass biomass was the highest at low altitude under both control and warming treatments (Figure S4B). The grass average coverage significantly increased after warming, except at low altitude (Table S3). The grass average height significantly increased following warming compared with the control (Table S4). The grass biomass showed a significant positive correlation with SOC (*p* < 0.035), TN (*p* < 0.045), and DOC (*p* < 0.045) contents at all four altitudes, and a negative correlation with soil DON (*p* < 0.037) and SM (*p* < 0.016) contents, except at low altitudes (Table S5).

### 3.2. Soil Fungal Alpha-Diversity

**Figure 2:**
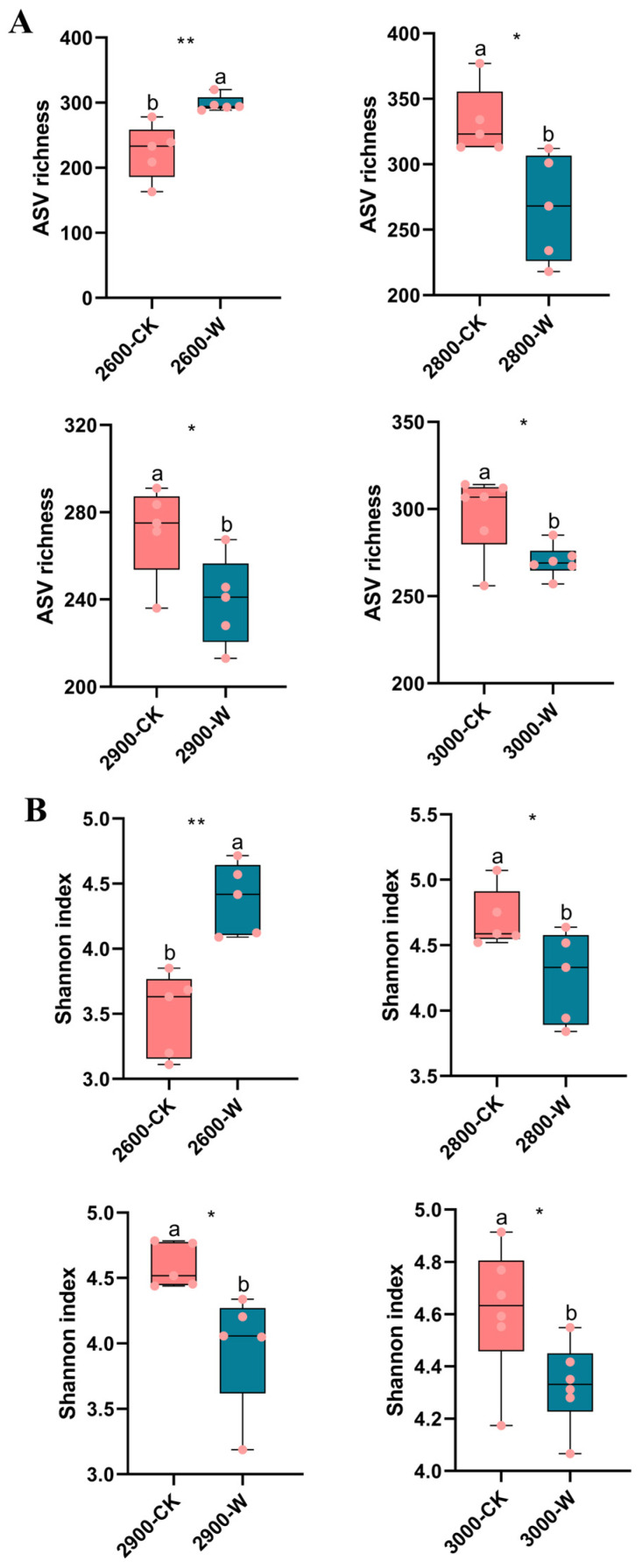
Effect of warming on soil fungal alpha-diversity along altitude gradients. (**A**) ASV richness; (**B**) Shannon index. Letters represent significant differences from paired-samples *t*-test analysis. *: *p* < 0.05; **: *p* < 0.01. ASV: Amplicon Sequence Variation. CK: control; W: warming. Low altitude (2600 masl), medium altitude (2800 masl), medium-high altitude (2900 masl), and high altitude (3000 masl).

A total of 1,991,770 fungal sequences were detected across all samples, with counts ranging from 28,174 to 56,410 sequences per sample. A total of 4457 fungal ASVs were detected across all samples, with counts ranging from 131 to 380 ASVs per sample. Warming significantly increased soil fungal alpha-diversity at low altitude, but decreased it at medium and high altitude compared with the control (Figure 2). The contrast patterns of fungal alpha-diversity were found along altitude gradients between control and warming treatments (Figure S5). At all four altitudes, the soil fungal alpha-diversity was significantly correlated with SOC content, DOC content, and grass biomass (*p* < 0.05 in all cases) (Table S6).

### 3.3. Soil Fungal Community Composition

**Figure 3:**
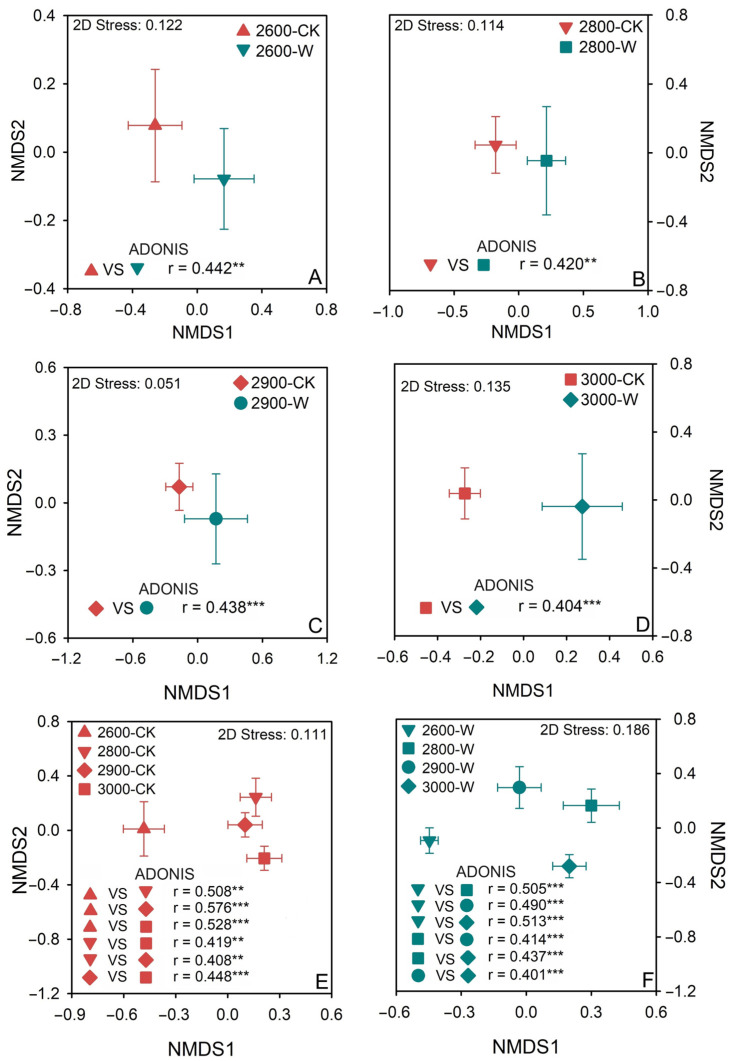
Fungal community composition indicated by non-metric multi-dimensional scaling (NMDS) and permutational MANOVA (ADONIS) at 2600 masl (**A**), 2800 masl (**B**), 2900 masl (**C**), 3000 masl (**D**), non-warming groups (**E**) and warming groups (**F**). CK: control; W: warming. Low altitude (2600 masl), medium altitude (2800 masl), medium-high altitude (2900 masl), and high altitude (3000 masl). **: *p* < 0.01; ***: *p* < 0.001.

Based on ADONIS and NMDS analysis, significant differences were observed in soil fungal community compositions between control and warming treatments at all four altitudes (Figure 3A–D). The fungal community composition showed significant differences along altitude gradients in both control and warming soils (Figure 3E,F). However, the differences in fungal community composition were larger along altitude gradients following warming compared with those for control soils (Figure 3E,F). Mantel test analysis showed that SOC content, DOC content, and grass biomass were the main factors affecting soil fungal community composition (Table S7).

The dominant soil fungal phyla were Ascomycota (70.66%), Basidiomycota (14.67%), and Mucoromycota (11.25%). Compared with the control, the warming treatment significantly reduced the relative abundance of Ascomycota except at low altitude (Figure S6A). Warming decreased the relative abundance of Basidiomycota at low and high altitudes but increased it at medium and medium-high altitudes (Figure S6B). The relative abundance of Mucoromycota increased in warming soils at all altitudes (Figure S6C). The relative abundance of Ascomycota, Basidiomycota, and Mucoromycota showed significant differences along altitude gradients in both control and warming soils (Figure S6D–F).

The main dominant genera in the soil were *Mortierella* (10.48%), *Thelebolus* (6.21%), and *Preussia* (3.66%) (Figure S7). The stamp analysis demonstrated that warming increased the relative abundance of *Mortierella* and *Nectria* but decreased the relative abundance of *Naganishia* and *Gaertneriomyces* at low altitudes (Figure S8A). Warming decreased the relative abundance of *Neoascochyta* and *Powellomyces* but increased the relative abundance of *Mortierella* and *Neopyrenochaeta* at medium altitude (Figure S8B). Warming increased the relative abundance of *Clavaria* and *Mortierella* and decreased the relative abundance of *Fusarium* at medium-high altitude (Figure S8C). Warming decreased the relative abundance of *Dactylonectria*, *Fusarium*, and *Neoascochyta* and increased the relative abundance of *Mortierella* at high altitude (Figure S8D).

The indicator analysis was used to identify fungal species associated with each treatment. The results showed three (*Podospora curvuloides*, *Preussia similis*, and *Hydnocystis japonica*) and three (*Preussia funiculata*, *Helvellosebacina helvelloides*, *Podospora vesticola*) indicator fungal species in control and warming soils at low altitude, respectively. Also, two (*Petrakia echinate*, *Leuconeurospora pulcherrima*) and two (*Clavaria falcata*, *Clavaria redoleoalii*) indicator fungal species were found in control and warming soils at medium altitude, respectively. Further, three (*Fusarium avenaceum*, *Leptosphaerulina australis*, *Fusarium oxysporum*) and two (*Camarophyllus borealis*, *Paraphaeosphaeria xanthorrhoeae*) indicator fungal species were detected in control and warming soils at medium-high altitude, respectively. Moreover, three (*Lachnellula fuscosanguinea*, *Naematelia aurantialba*, *Entrophospora infrequens*) and three (*Ustilago striiformis*, *Alatospora pulchella*, *Neosulcatispora agaves*) indicator fungal species were observed in control and warming soils at high altitude, respectively (Table S8).

### 3.4. Grass Pathogens and Soil Saprotrophs

**Figure 4:**
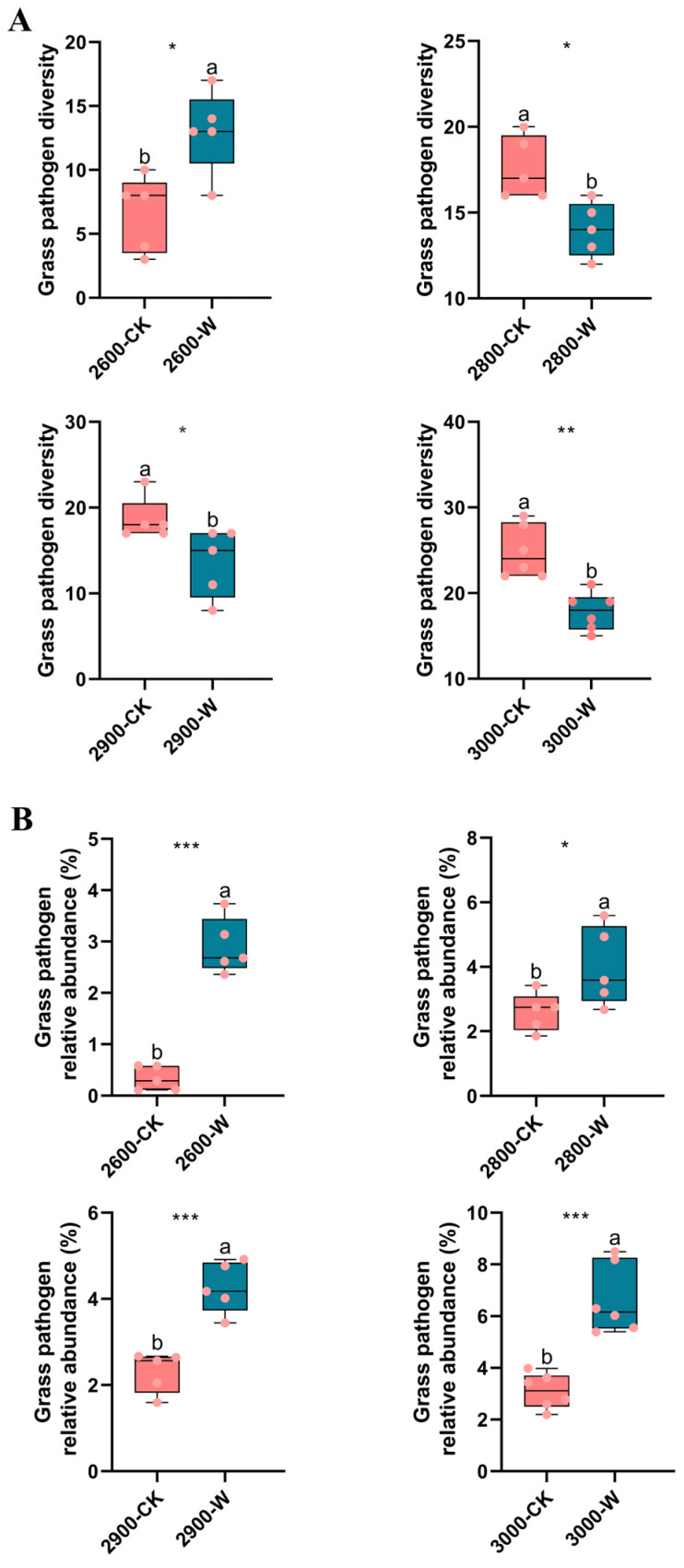
The diversity (**A**) and relative abundance (**B**) of grass pathogens along altitude gradients. Letters represent significant differences from paired-samples *t*-test analysis. *: *p* < 0.05; **: *p* < 0.01; ***: *p* < 0.001. CK: control; W: warming. Low altitude (2600 masl), medium altitude (2800 masl), medium-high altitude (2900 masl), and high altitude (3000 masl).

The potential grass pathogens and soil saprotrophs were identified using FUNGuild analysis. The grass pathogen diversity significantly decreased in warming soil compared with the control soil, except at low altitude (Figure 4A). However, the relative abundance of grass pathogens increased following warming at all four altitudes (Figure 4B). Both grass pathogen diversity and relative abundance displayed an increasing trend along altitude gradients in both control and warming soils (Figure S9). Warming also increased the relative abundance of dominant pathogens, such as *Oculimacula aestiva*, *Acicuseptoria rumicis*, *Septoria orchidearum*; *Paraphaeosphaeria xanthorrhoeae*, *Ustilago striiformis*, and *Ophiobolus cirsii* (Table S9). Soil saprotrophs might play an essential role in nutrient cycling. In this study, several soil saprotrophs were identified. Also, warming increased the relative abundance of soil saprotrophs, including *Preussia funiculate*, *Polyphilus sieberi*, *Trichosporiella cerebriformis*, and *Spirosphaera beverwijkiana* (Table S10).

### 3.5. Soil Fungal Network Analysis

The co-occurrence network analysis was used to model the interrelationships among soil fungal taxa at different altitudes. Most of the links in the network were associated with Ascomycota, Basidiomycota, Mucoromycota, and Chytridiomycota in control and warming soils at four altitudes. (Figure S10). The network diameter and the path length (average) decreased, whereas the clustering coefficient increased in warming soil compared with the control soil (Table S11). The network stability was evaluated based on natural connectivity. The results showed that the control soil had a higher fungal network stability than the warming soil at low altitude. In contrast, the warming soil had higher fungal network stability than the control soil at other altitudes (Figure S11). In control soils, the network stability was the highest at low altitude, while the network stability was the highest at high altitude following warming (Figure S11).

### 3.6. Soil Fungal Community Assembly Processes

**Figure 5:**
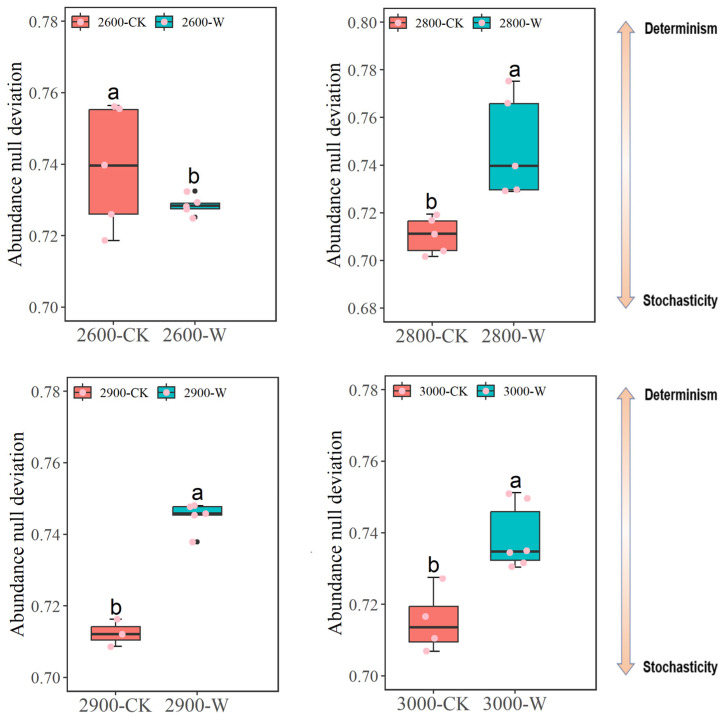
The fungal community assembly processes evaluated by the null deviation value (NDV) along altitude gradients. Letters represent significant differences from paired-samples *t*-test analysis. CK: control; W: warming. Low altitude (2600 masl), medium altitude (2800 masl), medium-high altitude (2900 masl), and high altitude (3000 masl).

The NDV (null deviation value) close to zero suggested that stochastic processes were more important in shaping community structure, whereas higher positive or negative null deviations suggested that deterministic processes were more important. The fungal community displayed a stochastic assembly process in warming soils compared with control soils at low altitudes, whereas it showed a deterministic process at medium and high altitudes (Figure 5).

## 4. Discussion

The subalpine meadows are sensitive to climatic changes [36]. Temperature is a limiting factor for the growth of subalpine grasslands [37]. The temperature increases following warming, thereby reducing the pressure of low temperatures on the grass growth in a subalpine ecosystem. Thus, the grass biomass, community height, and coverage increased following warming, which was consistent with the findings of other studies [38,39,40]. However, the grass diversity increased following warming (Figure 1A), which was inconsistent with previous findings, showing that warming decreased grass diversity in an alpine meadow [41,42]. This discrepancy might be due to the responses of different ecosystems (i.e., alpine and subalpine meadows) to warming. The soil carbon content increased following warming (Figure S2). Warming facilitated plant growth, inducing more organic carbon into soils by root exudates, dead branches, and leaves [43,44]. The nitrogen content did not change significantly, but AP content significantly decreased following warming (Figure S2G). This indicated that soil phosphorus rather than nitrogen might be the limited nutrient for grass growth in subalpine meadows.

Warming can directly or indirectly affect soil microbial communities [45,46]. Previous studies showed that soil microorganisms were sensitive to temperature shifts [47,48,49]; thus, warming could directly affect fungal community composition [50,51]. In addition, warming influenced aboveground vegetation growth, resulting in more grass biomass as observed in this study. Increased grass biomass induced abundant soil carbon resources. Also, the soil fungal community composition showed a primary correlation with SOC and DOC contents (Table S7). Thus, the changes in vegetation regulated soil properties, indirectly impacting fungal community composition [52]. However, the contrasting responses of fungal community assembly to warming were found at different altitudes. Compared to control, soil fungal community assembly might suggest the existence of more stochastic processes at low altitudes but more deterministic processes at medium and high altitudes (Figure 5). The grass biomass significantly increased up to the highest levels following warming at low altitudes, with huge carbon resources being distributed into soils, which may suggest the conformation of a favorable environment for belowground microorganisms. The competition within fungal taxa was reduced. Thus, the predicted community assembly might show more stochastic processes with higher fungal diversity at low altitudes following warming (Figure 2 and Figure 5). However, the fungal diversity decreased at medium and high altitudes following warming (Figure 2). The environmental conditions were harsher at higher altitudes, which might increase environmental filtering for soil fungal communities. Warming decreased SM, which might further enhance the community deterministic process (i.e., environmental filtering) to lower fungal diversity following warming at medium and high altitudes.

In this study, the network clustering coefficient increased and the average path length decreased in all warming soils (Table S11), suggesting a tighter network connection for material cycling and energy exchange within fungal taxa; this might improve the efficiency of soil nutrient cycling [53]. Ascomycota are important decomposers that help break down organic matter and promote material cycling [54,55]. Basidiomycota is related to the degradation of exogenous organic matter in soil and plays an important role in the degradation of plant residues [56,57]. Mucoromycota form a symbiotic relationship with plants, providing nutrients to promote plant growth [58,59]. Consistent with the aforementioned results, the relative abundance of saprotrophs increased following warming (Table S10). Warming increased soil organic matter content, facilitating more efficient material turnover by soil fungi. The stability of the fungal community network was significantly enhanced following warming, except at low altitude (Figure S11). A more complex microbial network showed significant resistance to disturbances and supported higher ecosystem multifunctionality. The ecosystems at high altitude might suffer more disturbances compared with those at low altitude [60]. Thus, the fungal community network was associated with more stability to cope with adverse environments in higher-altitude subalpine meadow ecosystems.

This study focused on grass pathogens following warming in a subalpine meadow ecosystem. The pathogen diversity showed similar patterns to the overall fungal diversity (Figure 2 and Figure 4A). The relative abundance of pathogens increased following warming (Figure 4B). Furthermore, the dominant grass pathogens were analyzed. The relative abundance of certain pathogens significantly increased following warming (Table S9). These pathogens caused black spots, root rot, and yellow spots in grasses [61,62,63,64,65,66]. The results indicated that warming led to the growth of specific pathogens, thereby increasing the probability of grass diseases and posing potential harm to subalpine grassland ecosystems.

## 5. Conclusions

### 5.1. The Effect of Warming on Aboveground Grass and Soil Organic Carbon Content

This study investigated the impact of warming on subalpine meadow ecosystems along altitude gradients. The temperature increases following warming, reducing the pressure of low temperatures on the grass growth by increasing grass biomass, community height, and coverage in the subalpine ecosystem. Warming facilitated plant growth, inducing more organic carbon into soils by root exudates, dead branches, and leaves to increase soil carbon content.

### 5.2. The Effect of Warming on Belowground Fungal Community

The results showed that warming significantly altered soil fungal community structure. However, the responses of soil fungal diversity and community assembly processes to warming varied between low and high altitudes. Warming triggered more stochastic processes at low altitudes, leading to an increase in fungal diversity. In contrast, warming induced more deterministic processes, enhancing environmental filtering and reducing fungal diversity at medium and high altitudes. Warming might improve the efficiency of soil nutrient cycling and organic matter turnover by increasing the relative abundance of dominant saprotrophs and improving network connectivity.

### 5.3. The Effect of Warming on Soil Pathogen

The relative abundance of pathogens increased following warming, posing potential risks to subalpine grassland ecosystems. The long-term monitoring of the effects of warming is essential, especially for pathogen-related shifts in subalpine meadow ecosystems.

## Data Availability

The raw data were submitted to the Sequence Read Archive (SRA) of NCBI under the accession number SRR29055324-SRR29055331.

## Supplementary Materials

The following supporting information can be downloaded at: https://www.mdpi.com/article/10.3390/microorganisms12122527/s1, Table S1: The temperature of air and soil between control and warming along altitude gradients; Table S2: Dominant grass species under different treatments; Table S3: Grass average coverage degree under different treatments along different altitude gradients; Table S4: Grass average height under different treatments along different altitude gradients; Table S5: Spearman correlations between grass biomass and soil biochemical properties along different altitude gradients; Table S6: Spearman correlations between fungal alpha-diversity, soil biochemical properties and grass biomass along different altitude gradients; Table S7: Mantel test showing the correlation between fungal community composition, soil biochemical properties and grass biomass along different altitude gradients; Table S8: Indicator analysis showing dominant indicator species of soil fungal species under different treatments along different altitude gradients; Table S9: Effect of warming on the relative abundance of dominant pathogens under different treatments along different altitude gradients; Table S10: Effect of warming on the relative abundance of soil saprotrophs under different treatments along different altitude gradients; Table S11: Topological properties of soil–fungal interaction networks under different treatments along different altitude gradients. Figure S1: Open-top chamber for warming in the field; Figure S2: Effect of warming on soil properties under different treatments along different altitude gradients. Letters indicate significant differences based on paired-samples *t*-test analysis; Figure S3: Soil properties under different treatments along different altitude gradients. Letters indicate significant differences based on a one-way analysis of variance (ANOVA, *p* < 0.05); Figure S4: Aboveground grass diversity (A) and grass biomass (B) under different treatments along different altitude gradients; Figure S5: Alpha-diversity under different treatments along different altitude gradients; Figure S6: Relative abundance of Ascomycota, Basidiomycota and Mucoromycota under different treatments along different altitude gradients; Figure S7: Dominant fungal genera under different treatments along different altitude gradients; Figure S8: Effect of warming on the relative abundance of fungal genera; Figure S9: Grass pathogen diversity (A) and relative abundance (B) under different soil treatments along different altitude gradients; Figure S10: Co-occurrence network structure of soil fungal community; Figure S11: Stability of fungal co-occurrence network.
