# Supplementary material for: Effect of Warming on Soil Fungal Community Along Altitude Gradients in a Subalpine Meadow
Source: Microorganisms. 2024 Dec 7;12(12):2527. doi: 10.3390/microorganisms12122527 (PMC11678067; doi:10.3390/microorganisms12122527)
Supplement: Supplementary file 1 [file microorganisms-12-02527-s001.zip › microorganisms-3344977-supplementary.pdf]

Supporting Information

Table S1. The temperature of air and soil between control and warming along altitude gradients. CK: Control; W: warming. Low altitude (2600 masl), medium altitude (2800 masl), medium-high altitude (2900 masl), and high altitude (3000 masl).

**Altitude (masl)**	**Air temperature (ºC)**			**Soil temperature (ºC)**		
	**Control**	**Warming**		**Control**	**Warming**	
	14.5	16.8	**+2.3**	3.2	5.6	**+2.4**
	13.6	15.3	**+1.7**	3.7	5.4	**+1.7**
2600	14.4	17.0	**+2.6**	3.8	5.4	**+1.6**
	14.8	17.2	**+2.4**	3.3	5.3	**+2.0**
	13.2	16.1	**+2.9**	3.6	6.0	**+2.4**
	12.1	15.1	**+3.0**	1.9	4.5	**+2.6**
	12.2	15.0	**+2.8**	2.4	5.8	**+3.3**
2800	12.5	15.5	**+3.0**	1.7	4.7	**+3.0**
	12.2	15.3	**+3.2**	1.5	4.5	**+3.0**
	12.1	15.0	**+2.9**	2.1	5.4	**+3.3**
	12.5	15.5	**+3.0**	1.8	4.3	**+2.4**
	12.6	15.8	**+3.2**	2.0	3.6	**+1.6**
2900	11.5	14.5	**+3.0**	1.5	3.7	**+2.2**
	11.7	14.9	**+3.2**	1.5	3.3	**+1.9**
	11.3	14.3	**+3.0**	1.4	3.9	**+2.5**
	9.9	12.9	**+3.0**	1.5	4.2	**+2.7**
	9.8	12.7	**+2.9**	1.4	3.3	**+1.9**
3000	9.7	12.7	**+3.0**	1.6	3.1	**+1.5**
	9.7	12.8	**+3.1**	1.5	3.3	**+1.9**
	9.5	12.9	**+3.4**	1.3	3.3	**+1.9**

Note: The temperature was measured on 13 September 2023.

Table S2. Dominant grass species under different treatments.

Site	Coordinates	Altitude (masl)	Dominant taxa
2600-CK	39° 3′53″ N<br>113° 32′30″ E	2600	*Viola philippica, Rumex acetosa*, *Lablab purpureus*, *Thalictrum alpinum*, *Geranium wilfordii Parnassia palustris*, *Silene conoidea*, *Ranunculus tanguticus*, *Gentiana macrophylla*, *Carex alatauensis*, *Carex* sp., and *Polygonum viviparum*
2600-W	39° 3′53″ N<br>113° 32′30″ E	2600	*Leontopodium leontopodioides*, *Taraxacum mongolicum*, *Viola philippica*, *Rumex acetosa*, *Polygonum viviparum*, *Poa annua*, *Dianthus superbus*, *Pedicularis tatarinowii*, *Silene conoidea*, *Geranium wilfordii*, *Parnassia palustris*, *Carex alatauensis*, *Carex* sp., and *Ranunculus tanguticus*
2800-CK	39° 4′1.21″ N<br>113° 32′56″ E	2800	*Viola philippica, Carex alatauensis*, *Carex* sp., *Ranunculus tanguticus*, *Leontopodium leontopodioides*, *Taraxacum mongolicum*, *Potentilla fragarioides*, *Polygonum viviparum*, *Astragalus membranaceus*, *Gentiana scabra*, and *Thalictrum petaloideum*
2800-W	39° 4′1.21″ N<br>113° 32′55″ E	2800	*Carex alatauensis*, *Carex* sp., *Ranunculus tanguticus*, *Taraxacum mongolicum*, *Polygonum viviparum*, *Potentilla fragarioides*, *Ranunculus tanguticus*, *Carum buriaticum*, *Anaphalis sinica*, *Astragalus membranaceus*, *Thalictrum petaloideum*, *Viola biflora*, *Poa annua*, *Saussurea purpurascens,* and *Viola philippica*

Continued Table S2

Site	Coordinates	Altitude (masl)	Dominant taxa
2900-CK	39° 4′32.243″ N<br>113° 33′30″ E	2900	*Viola philippica, Carex alatauensis*, *Carex* sp., *Anaphalis sinica*, *Leontopodium leontopodioides*, *Polygonum viviparum*, *Thalictrum petaloideum*, *Potentilla fragarioides*, *Talinum paniculatum*, *Saussurea purpurascens*, and *Ranunculus tanguticus*
2900-W	39° 4′32.243″ N<br>113° 33′30″ E	2900	*Viola philippica, Carex alatauensis*, *Carex* sp., *Taraxacum mongolicum*, *Polygonum viviparum*, *Potentilla fragarioides*, *Saussurea purpurascens*, *Festuca rubra*, *Anaphalis sinica*, *Heteropappus altaicus*, *Thalictrum petaloideum*, *Leontopodium leontopodioides*, *Talinum paniculatum*, and *Oreomecon nudicaulis*
3000-CK	39° 4′51.312″ N<br>113° 33′45″ E	3000	*Carex alatauensis*, *Carex* sp., *Saussurea purpurascens*, *Oxytropis caerulea*, *Thalictrum petaloideum*, *Polygonum viviparum*, *Potentilla fragarioides*, *Astragalus membranaceus,* and *Viola philippica*
3000-W	39° 4′51.312″ N<br>113° 33′45″ E	3000	*Carex alatauensis*, *Carex* sp., *Saussurea purpurascens*, *Polygonum viviparum*, *Potentilla fragarioides*, *Thalictrum petaloideum*, *Astragalus Viola biflora*, *Oxytropis caerulea*, *Festuca rubra*, *Leontopodium leontopodioides,* and *Viola philippica*

CK: Control; W: warming. Low altitude (2600 masl), medium altitude (2800 masl), medium-high altitude (2900 masl), and high altitude (3000 masl).

Table S3. Grass average coverage degree under different treatments along different altitude gradients.

Altitude (masl)	Grass coverage degree (%)		
	CK	W	*P*
2600	100 ± 0	100 ± 0	1.000
2800	93 ± 3	100 ± 0	**0.016**
2900	90 ± 5	100 ± 0	**0.036**
3000	80 ± 5	98 ± 2	**0.005**

CK: Control; W: warming. Low altitude (2600 masl), medium altitude (2800 masl), medium-high altitude (2900 masl), and high altitude (3000 masl).

Table S4. Grass average height under different treatments along different altitude gradients.

Altitude (masl)	Grass average height (cm)		
	CK	W	*P*
2600	12.09 ± 3.47	21.58 ± 2.48	**0.018**
2800	8.76 ± 4.50	19.07 ± 1.42	**0.019**
2900	9.57 ± 1.54	18.31 ± 1.62	**0.003**
3000	5.53 ± 0.65	16.14 ± 4.79	**0.019**

CK: Control; W: warming. Low altitude (2600 masl), medium altitude (2800 masl), medium-high altitude (2900 masl), and high altitude (3000 masl).

Table S5. Spearman correlations between grass biomass and soil biochemical properties along different altitude gradients.

Soil biochemical properties	Grass biomass (g/plot)							
	2600 masl		2800 masl		2900 masl		3000 masl	
	R	*P*	R	*P*	R	*P*	R	*P*
PH	-0.270	0.451	-0.031	0.932	-0.290	0.417	-0.424	0.169
SOC (g/kg)	**0.753***	**0.012**	**0.839****	**0.002**	**0.668***	**0.035**	**0.745****	**0.005**
TN (g/kg)	**0.739***	**0.015**	**0.660***	**0.038**	**0.643***	**0.045**	**0.831*****	**0.001**
TP (%)	0.300	0.400	0.359	0.308	-0.056	0.878	-0.379	0.225
NH_4^+-N (mg/kg)	-0.422	0.225	0.053	0.885	-0.029	0.937	-0.271	0.394
AP (mg/kg)	-0.377	0.282	-0.483	0.157	**-0.720**	**0.019**	-0.246	0.441
NO_3^--N (mg/kg)	-0.072	0.843	-0.365	0.300	-0.035	0.924	-0.014	0.966
DOC (mg/kg)	**0.731***	**0.016**	**0.655***	**0.040**	**0.644***	**0.045**	**0.900*****	**0.000**
DON (mg/kg)	**0.726***	**0.017**	**-0.874*****	**0.001**	**-0.662***	**0.037**	**-0.653***	**0.021**
SM (%)	-0.329	0.353	**-0.741***	**0.014**	**-0.766***	**0.010**	**-0.675***	**0.016**

*: $P < 0.05$; **: $P < 0.01$; ***: $P < 0.001$. Low altitude (2600 masl), medium altitude (2800 masl), medium-high altitude (2900 masl), and high altitude (3000 masl).

Table S6. Spearman correlations between fungal alpha-diversity, soil biochemical properties and grass biomass along different altitude gradients.

**Variables**	2600 masl		2800 masl		2900 masl		3000 masl	
	ASV	Shannon	ASV	Shannon	ASV	Shannon	ASV	Shannon
pH	–0.261	–0.321	0.055	0.319	0.297	0.479	0.559	0.319
SOC (g/kg)	**0.842****	**0.770****	**–0.729***	**–0.662***	**–0.648***	**–0.685***	**–0.720****	**–0.701***
TN (g/kg)	**0.818****	**0.733***	**–0.699***	–0.179	–0.036	–0.401	–0.671*	–0.179
TP (%)	0.500	0.250	–0.326	0.218	–0.049	–0.270	–0.155	0.218
NH_4^+-N (mg/kg)	**–0.723***	**–0.839****	–0.188	0.364	–0.188	–0.079	–0.014	0.364
AP (mg/kg)	–0.280	–0.316	0.427	0.056	0.309	0.770**	–0.070	0.056
NO_3^--N (mg/kg)	–0.212	–0.115	0.455	–0.298	–0.224	–0.091	–0.252	–0.298
DOC (mg/kg)	**0.745***	**0.624***	**–0.697***	**–0.564***	**–0.705***	**-0.845****	**–0.678***	**–0.564***
DON (mg/kg)	**0.721***	**0.709***	**0.657***	0.431	0.079	**0.648***	0.483	0.431
SM (%)	0.146	–0.164	**0.879****	0.235	0.321	0.612	0.217	0.235
Grass biomass (g/plot)	**0.758***	**0.867****	**–0.685***	**–0.792***	**–0.762***	**–0.661***	**–0.769****	**–0.736****

*: $P < 0.05$; **: $P < 0.01$. ASV: Amplicon sequence variant; Shannon: Shannon index. Low altitude (2600 masl), medium altitude (2800 masl), medium-high altitude (2900 masl), and high altitude (3000 masl).

Table S7. Mantel test showing the correlation between fungal community composition, soil biochemical properties and grass biomass along different altitude gradients.

**Variables**	2600 masl		2800 masl		2900 masl		3000 masl	
	R	*P*	R	*P*	R	*P*	R	*P*
pH	0.017	0.449	–0.298	0.932	0.193	0.100	–0.012	0.572
SOC (g/kg)	**0.232***	**0.050**	**0.507***	**0.025**	**0.251***	**0.044**	**0.218***	**0.032**
TN (g/kg)	**0.282***	**0.044**	0.348	0.090	0.151	0.217	–0.019	0.605
TP (%)	–0.049	0.609	0.224	0.129	–0.141	0.814	–0.033	0.587
NH_4^+-N (mg/kg)	0.212	0.085	–0.441	0.993	–0.184	0.847	0.118	0.175
AP (mg/kg)	0.189	0.132	0.089	0.366	0.089	0.302	0.171	0.059
NO_3^--N (mg/kg)	–0.075	0.677	0.314	0.052	0.099	0.300	0.024	0.447
DOC (mg/kg)	0.156	0.175	**0.455***	**0.050**	**0.306*****	**0.001**	**0.272***	**0.008**
DON (mg/kg)	0.080	0.281	0.100	0.310	0.092	0.300	**0.200***	**0.032**
SM (%)	0.170	0.125	**0.376***	**0.049**	**0.288***	**0.030**	0.115	0.166
Grass biomass (g/plot)	**0.440***	**0.013**	0.091	0.271	**0.341****	**0.003**	**0.331****	**0.005**

*: $P < 0.05$; **: $P < 0.01$; ***: $P < 0.001$. Low altitude (2600 masl), medium altitude (2800 masl), medium-high altitude (2900 masl), and high altitude (3000 masl).

Table S8. Indicator analysis showing dominant indicator species of soil fungal species under different treatments along different altitude gradients.

	Indicator value	*P*	Taxonomy	Relative abundance (%)
**2600-CK**	0.495	0.016	s__*Podospora curvuloides*	23.40
	0.676	0.001	s__*Preussia similis*	8.916
	0.741	0.002	s__*Hydnocystis japonica*	6.062
**2600-W**	0.582	0.009	s__*Preussia funiculata*	21.37
	0.729	0.009	s__*Helvellosebacina helvelloides*	10.99
	0.632	0.004	s__*Podospora vesticola*	9.601
**2800-CK**	0.341	0.043	s__*Petrakia echinata*	5.821
	0.414	0.035	s__*Leuconeurospora pulcherrima*	1.697
**2800-W**	0.446	0.003	s__*Clavaria falcata*	16.00
	0.476	0.007	s__*Clavaria redoleoalii*	1.051
**2900-CK**	0.240	0.034	s__*Fusarium avenaceum*	71.61
	0.802	0.023	s__*Leptosphaerulina australis*	68.85
	0.251	0.024	s__*Fusarium oxysporum*	33.60
**2900-W**	0.469	0.022	s__*Camarophyllus borealis*	10.65
	0.413	0.047	s__ *Paraphaeosphaeria xanthorrhoeae*	6.055
**3000-CK**	0.539	0.046	s__*Lachnellula fuscosanguinea*	32.98
	0.446	0.050	s__*Naematelia aurantialba*	11.61
	0.366	0.046	s__*Entrophospora infrequens*	7.306
**3000-W**	0.806	0.001	s__*Ustilago striiformis*	6.179
	0.421	0.016	s__*Alatospora pulchella*	5.867
	0.647	0.001	s__ *Neosulcatispora agaves*	3.393

CK: Control; W: warming. Low altitude (2600 masl), medium altitude (2800 masl), medium-high altitude (2900 masl), and high altitude (3000 masl).

Table S9. Effect of warming on the relative abundance of dominant pathogens under different treatments along different altitude gradients.

Altitude (masl)	Dominant pathogen relative abundance (%)			
	Taxonomy	CK	W	*P*
**2600**	**s__*Oculimacula aestiva***	**0.028 ± 0.577[b]**	**0.852 ± 0.731[a]**	**0.036**
	s__*Botrytis porri*	0.000 ± 0.000[a]	0.285 ± 0.638[a]	0.347
	**s__*Acicuseptoria rumicis***	**0.000 ± 0.000[b]**	**0.254 ± 0.241[a]**	**0.046**
	s__*Seimatosporium pistaciae*	0.001 ± 0.003[a]	0.216 ± 0.238[a]	0.078
**2800**	s__*Oculimacula aestiva*	0.900 ± 0.830[a]	0.792 ± 0.847[a]	0.843
	**s__*Septoria orchidearum***	**0.125 ± 0.126[b]**	**0.915 ± 0.723[a]**	**0.043**
	s__*Ophiobolus cirsii*	0.149 ± 0.307[a]	0.242 ± 0.195[a]	0.581
	s__*Paraleptosphaeria macrospora*	0.176 ± 0.217[a]	0.184 ± 0.257[a]	0.956
**2900**	**s__*Paraphaeosphaeria xanthorrhoeae***	**0.110 ± 0.186[b]**	**1.085 ± 0.787[a]**	**0.027**
	s__*Muriphaeosphaeria viburni*	0.036 ± 0.082[a]	0.465 ± 0.882[a]	0.311
	s__*Ophiobolus cirsii*	0.121 ± 0.179[a]	0.243 ± 0.191[a]	0.326
	s__*Dendrophoma juglandina*	0.000 ± 0.000[a]	0.400 ± 0.553[a]	0.144
**3000**	**s__*Ustilago striiformis***	**0.024 ± 0.060[b]**	**0.990 ± 0.970[a]**	**0.035**
	s__*Oculimacula acuformis*	0.202 ± 0.202[a]	0.188 ± 0.142[a]	0.892
	s__*Oculimacula aestiva*	0.181 ± 0.206[a]	0.186 ± 0.137[a]	0.936
	**s__*Ophiobolus cirsii***	**0.000 ±0.000[b]**	**0.150 ± 0.161[a]**	**0.046**

Superscript letters on numbers indicate significant differences based on paired-samples *t*-test analysis. CK: Control; W: warming. Low altitude (2600 masl), medium altitude (2800 masl), medium-high altitude (2900 masl), and high altitude (3000 masl).

Table S10. Effect of warming on the relative abundance of soil saprotrophs under different treatments along different altitude gradients.

Altitude (masl)	Dominant saprotroph relative abundance (%)			
	Taxonomy	CK	W	*P*
**2600**	**s__*Preussia funiculata***	**1.214 ± 1.294[b]**	**2.886 ± 2.315[a]**	**0.042**
	s__*Podospora curvuloides*	2.262 ± 1.326[a]	2.299 ± 3.997[a]	0.983
	s__*Podospora vesticola*	0.350 ± 0.334[a]	1.850 ± 1.434[a]	0.138
**2800**	s__*Rutstroemia paludosa*	0.097 ± 0.150[a]	0.402 ± 0.633[a]	0.234
	**s__*Polyphilus sieberi***	**1.047 ± 0.286[b]**	**2.005 ± 0.725[a]**	**0.049**
	s__*Trichopeziza sulphurea*	0.017 ± 0.035[a]	0.652 ± 1.418[a]	0.337
**2900**	**s__*Trichosporiella cerebriformis***	**0.216 ± 0.048[b]**	**0.431 ± 0.125[a]**	**0.040**
	s__*Gyoerffyella craginiformis*	2.432 ± 1.760[a]	1.177 ± 2.175[a]	0.463
	s__*Polyphilus sieberi*	0.766 ± 0.498[a]	1.413 ± 1.083[a]	0.102
**3000**	s__*Polyphilus sieberi*	3.315±1.438[a]	3.154 ± 1.073[a]	0.825
	**s__*Spirosphaera beverwijkiana***	**0.365 ± 0.210[b]**	**1.306 ± 0.841[a]**	**0.033**
	s__*Myrmecridium spartii*	0.007 ± 0.012[a]	0.185 ± 0.411[a]	0.343

Superscript letters on numbers indicate significant differences based on paired-samples *t*-test analysis. CK: Control; W: warming. Low altitude (2600 masl), medium altitude (2800 masl), medium-high altitude (2900 masl), and high altitude (3000 masl).

Table S11. Topological properties of soil–fungal interaction networks under different treatments along different altitude gradients.

Property	2600-CK	2600-W	2800-CK	2800-W	2900-CK	2900-W	3000-CK	3000-W
**Nodes**	147	106	168	158	150	161	192	168
**Edges**	2966	906	1872	2442	1754	2690	2946	3086
**Density**	0.138	0.081	0.067	0.098	0.078	0.104	0.080	0.110
**Modularity**	0.621	0.799	0.786	0.793	0.694	0.693	0.796	0.764
**Degree (average)**	20.177	8.547	11.143	15.456	11.693	16.708	15.344	18.369
**Network diameter**	9	4	7	6	9	4	15	6
**Clustering coefficient**	0.865	0.908	0.860	0.927	0.810	0.944	0.851	0.909
**Path length (average)**	2.065	1.280	1.483	1.392	2.469	1.297	3.905	1.424

CK: Control; W: warming. Low altitude (2600 masl), medium altitude (2800 masl), medium-high altitude (2900 masl), and high altitude (3000 masl).

Figure S1. Open-top chamber for warming in the field.

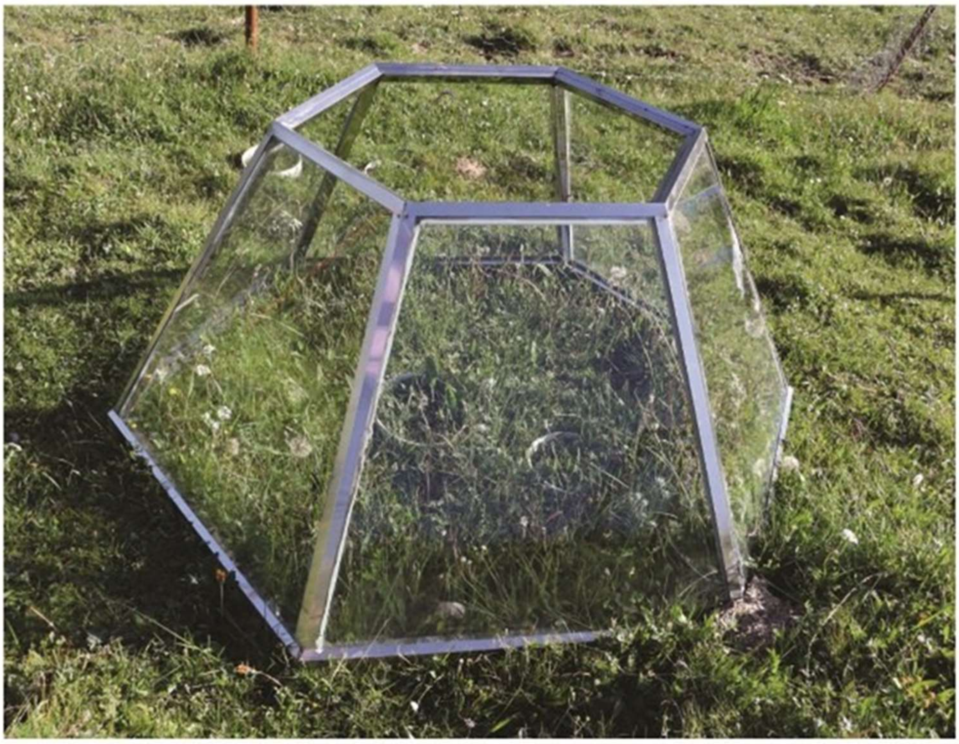

Figure S2. Effect of warming on soil properties under different treatments along different altitude gradients. Letters indicate significant differences based on paired-samples *t*-test analysis.

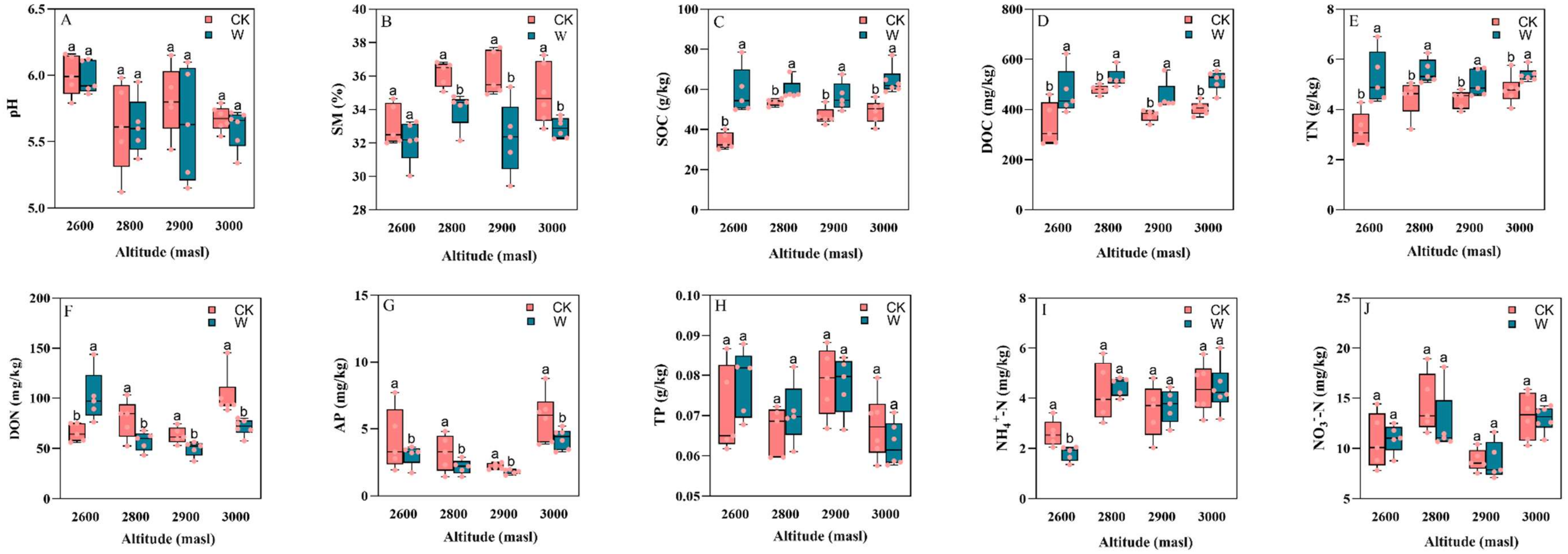

CK: Control; W: warming. Low altitude (2600 masl), medium altitude (2800 masl), medium-high altitude (2900 masl), and high altitude (3000 masl).

SM: soil moisture; SOC: soil organic carbon; DOC: dissolved organic carbon; TN: total nitrogen; DON: dissolved organic nitrogen; AP: Available phosphorous; TP: total phosphorus; NH_4^+-N: ammoniacal nitrogen; NO_3^--N: nitrate nitrogen.

Figure S3. Soil properties under different treatments along different altitude gradients. Letters indicate significant differences based on a one-way analysis of variance (ANOVA, $P < 0.05$).

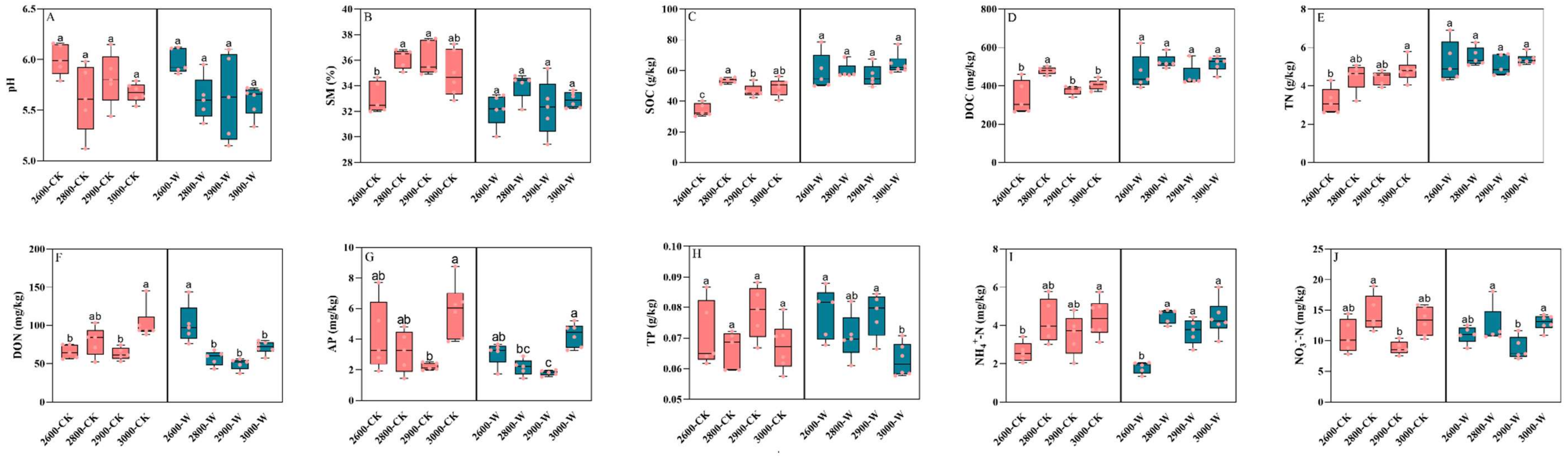

Low altitude (2600 masl), medium altitude (2800 masl), medium-high altitude (2900 masl), and high altitude (3000 masl).

SM: soil moisture; SOC: soil organic carbon; DOC: dissolved organic carbon; TN: total nitrogen; DON: dissolved organic nitrogen; AP: Available phosphorous; TP: total phosphorus; NH_4^+-N: ammoniacal nitrogen; NO_3^--N: nitrate nitrogen.

Figure S4. Aboveground grass diversity (A) and grass biomass (B) under different treatments along different altitude gradients.

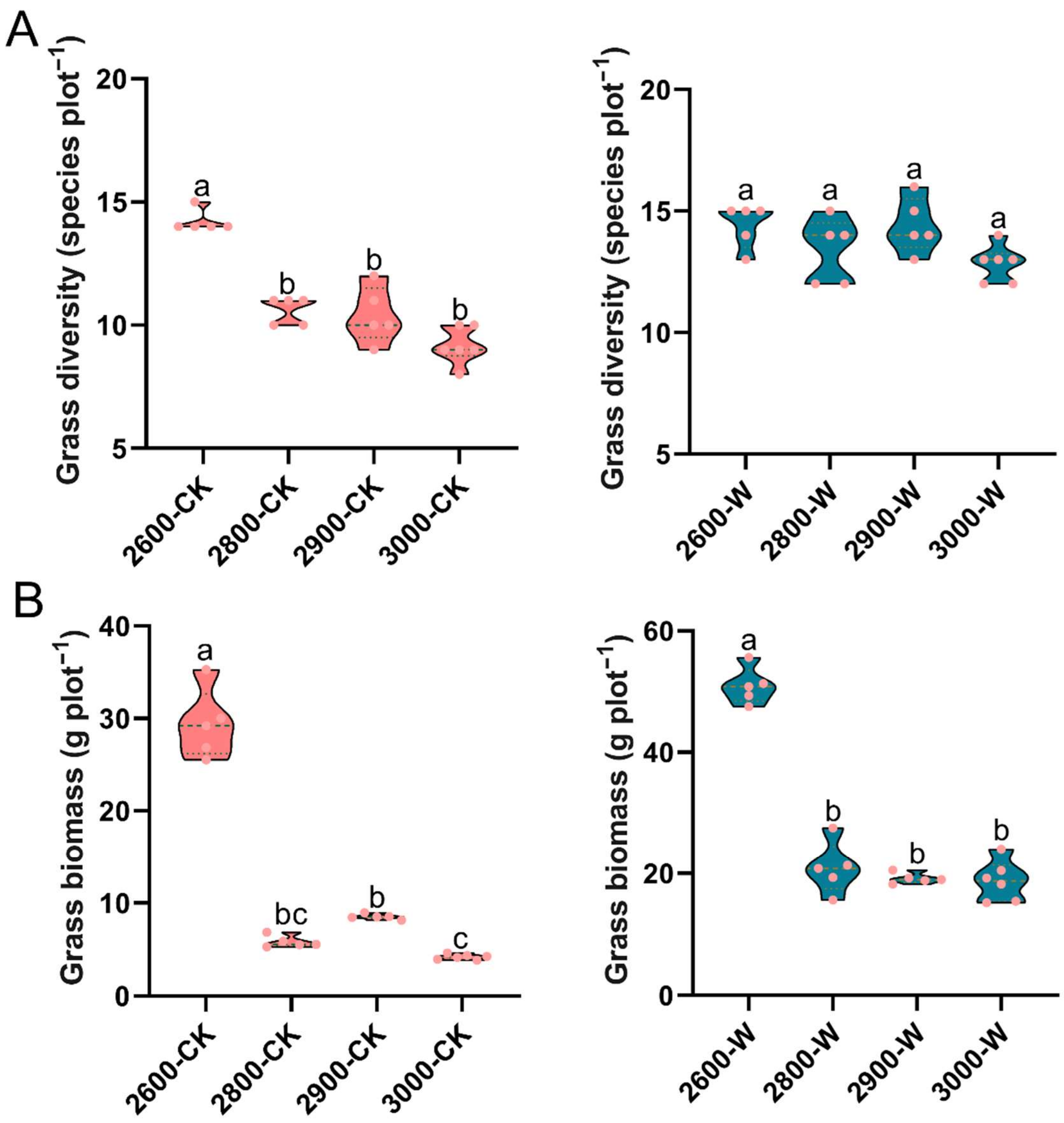

Letters indicate significant differences based on a one-way analysis of variance (ANOVA, $P < 0.05$). CK: Control; W: warming. Low altitude (2600 masl), medium altitude (2800 masl), medium-high altitude (2900 masl), and high altitude (3000 masl).

Figure S5. Alpha-diversity under different treatments along different altitude gradients.

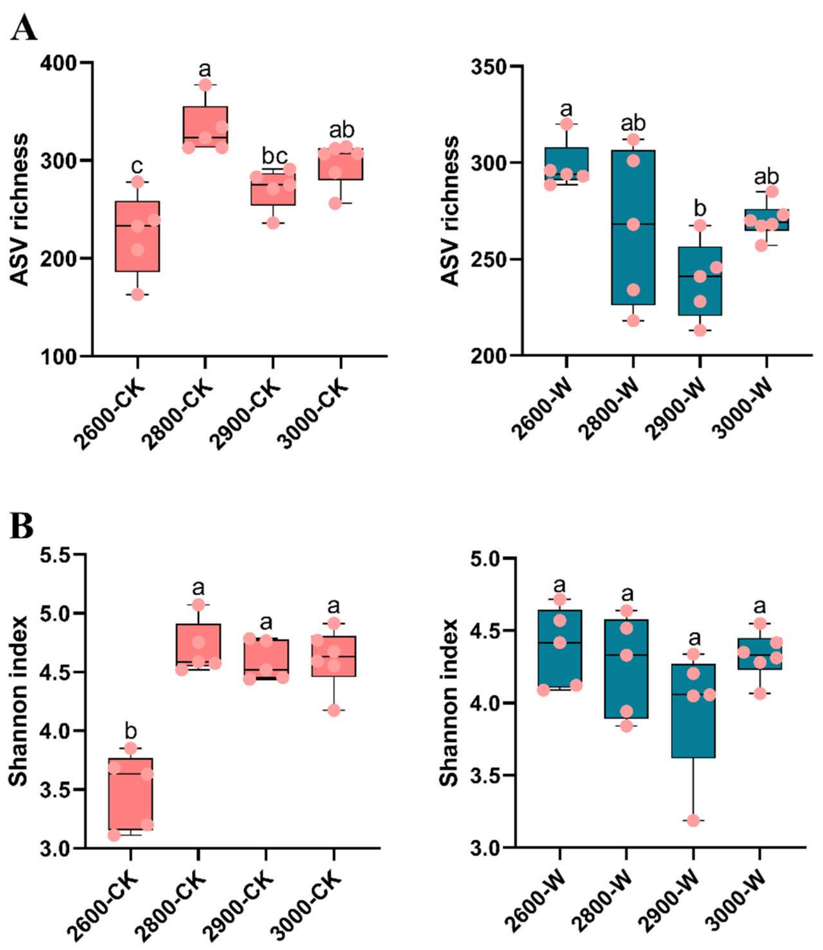

Letters indicate significant differences based on a one-way analysis of variance (ANOVA, $P < 0.05$). CK: Control; W: warming. Low altitude (2600 masl), medium altitude (2800 masl), medium-high altitude (2900 masl), and high altitude (3000 masl).

Figure S6. Relative abundance of Ascomycota, Basidiomycota and Mucoromycota under different treatments along different altitude gradients.

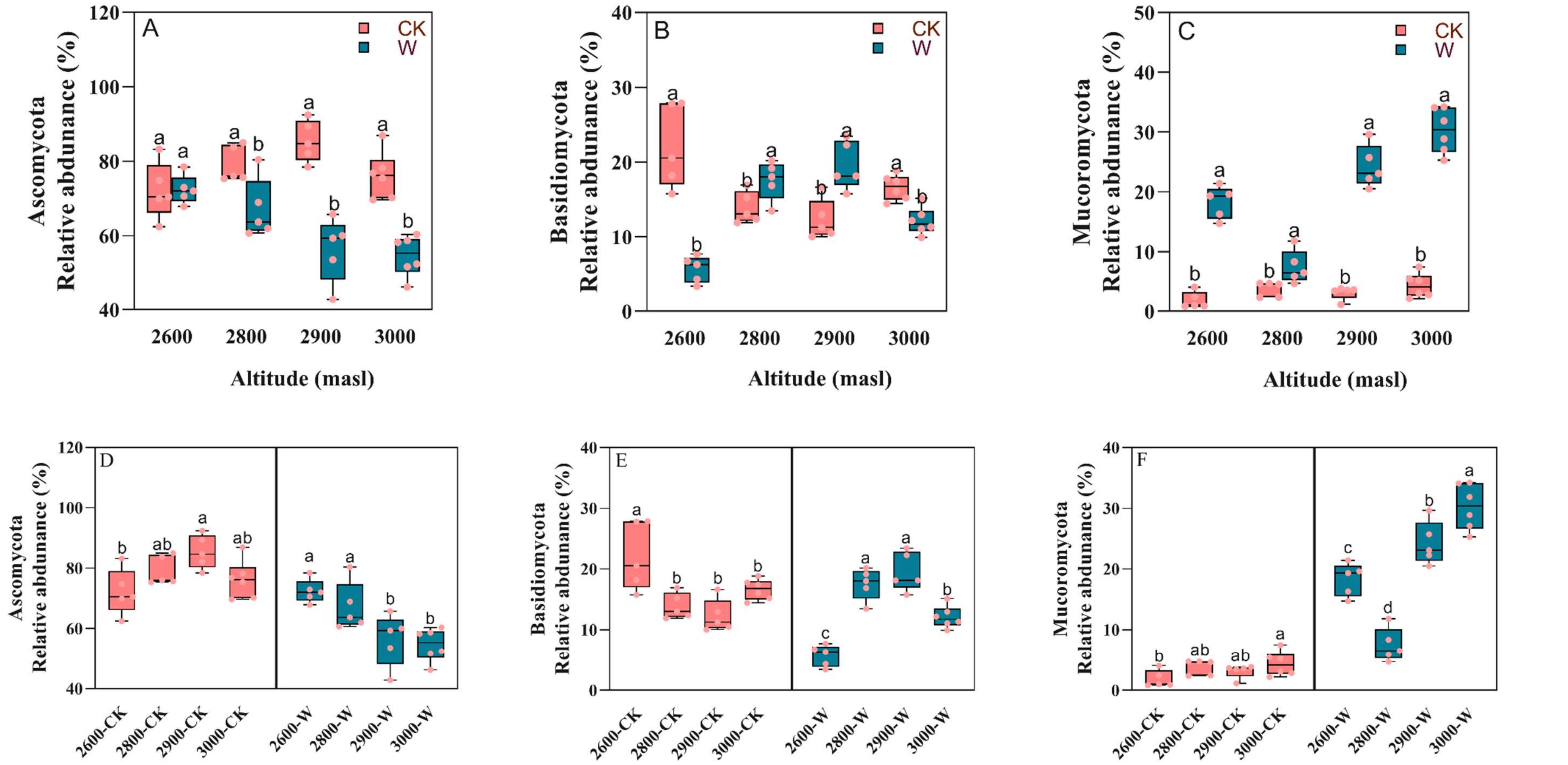

CK: Control; W: warming. Low altitude (2600 masl), medium altitude (2800 masl), medium-high altitude (2900 masl), and high altitude (3000 masl).

Figure S7. Dominant fungal genera under different treatments along different altitude gradients.

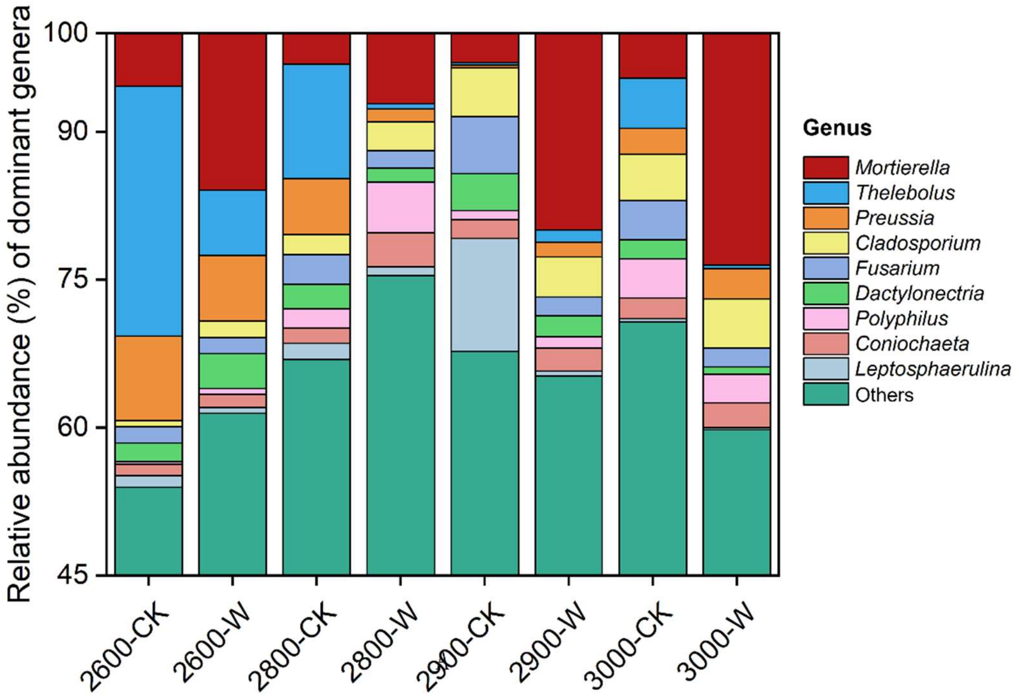

CK: Control; W: warming. Low altitude (2600 masl), medium altitude (2800 masl), medium-high altitude (2900 masl), and high altitude (3000 masl).

Figure S8. Effect of warming on the relative abundance of fungal genera.

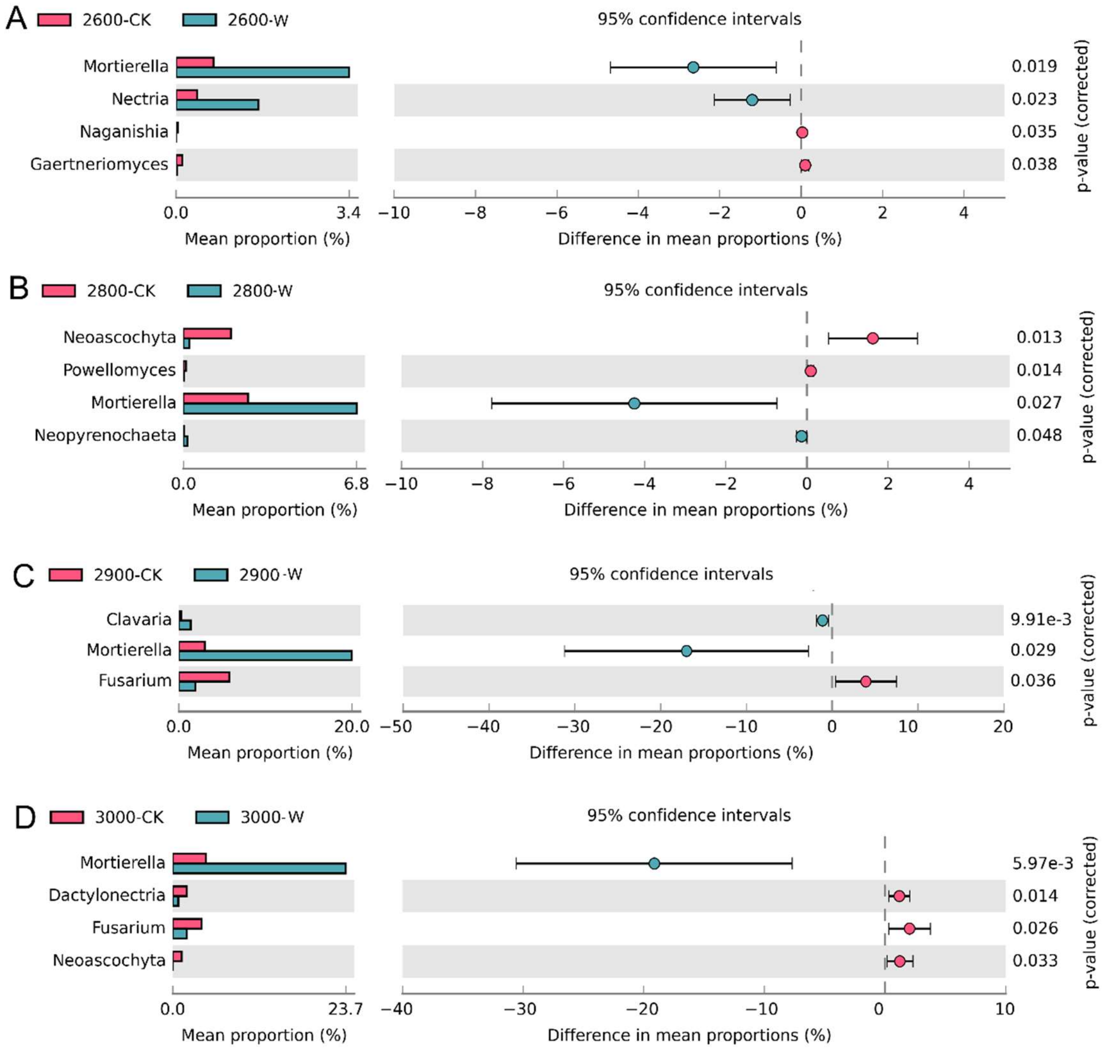

CK: Control; W: warming. Low altitude (2600 masl), medium altitude (2800 masl), medium-high altitude (2900 masl), and high altitude (3000 masl).

Figure S9. Grass pathogen diversity (A) and relative abundance (B) under different soil treatments along different altitude gradients.

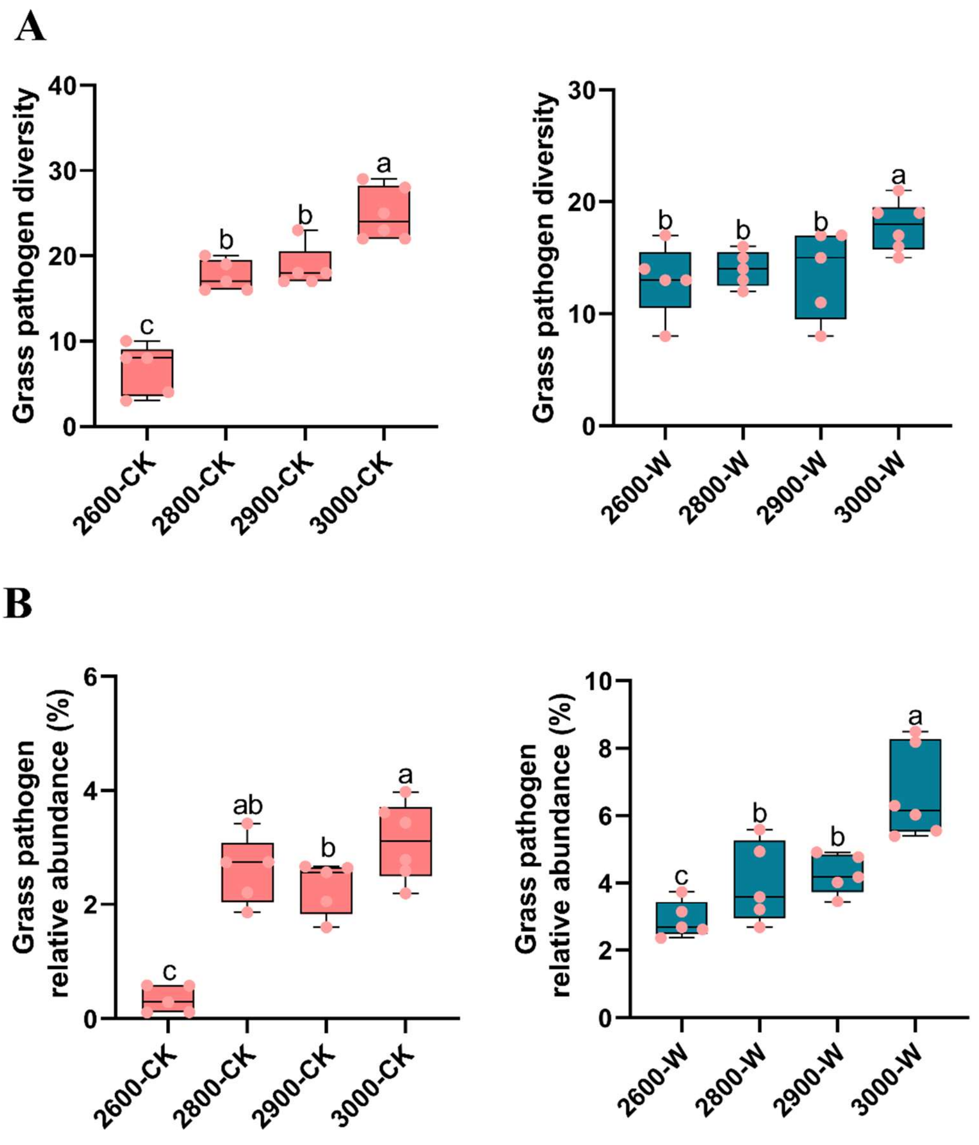

Letters indicate significant differences based on a one-way analysis of variance (ANOVA, $P < 0.05$). CK: Control; W: warming. Low altitude (2600 masl), medium altitude (2800 masl), medium-high altitude (2900 masl), and high altitude (3000 masl).

Figure S10. Co-occurrence network structure of soil fungal community.

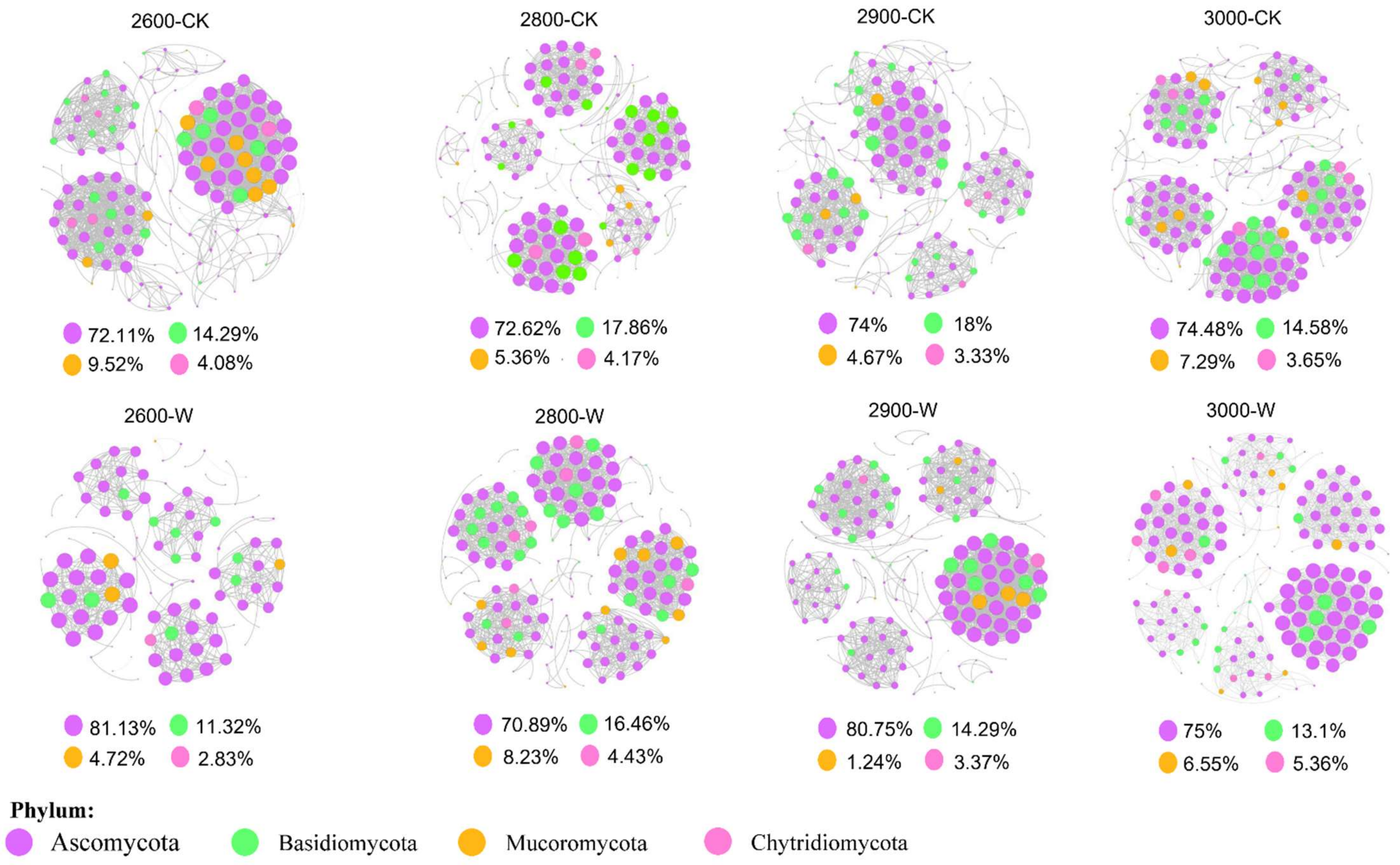

CK: Control; W: warming. Low altitude (2600 masl), medium altitude (2800 masl), medium-high altitude (2900 masl), and high altitude (3000 masl).

Figure S11. Stability of fungal co-occurrence network.

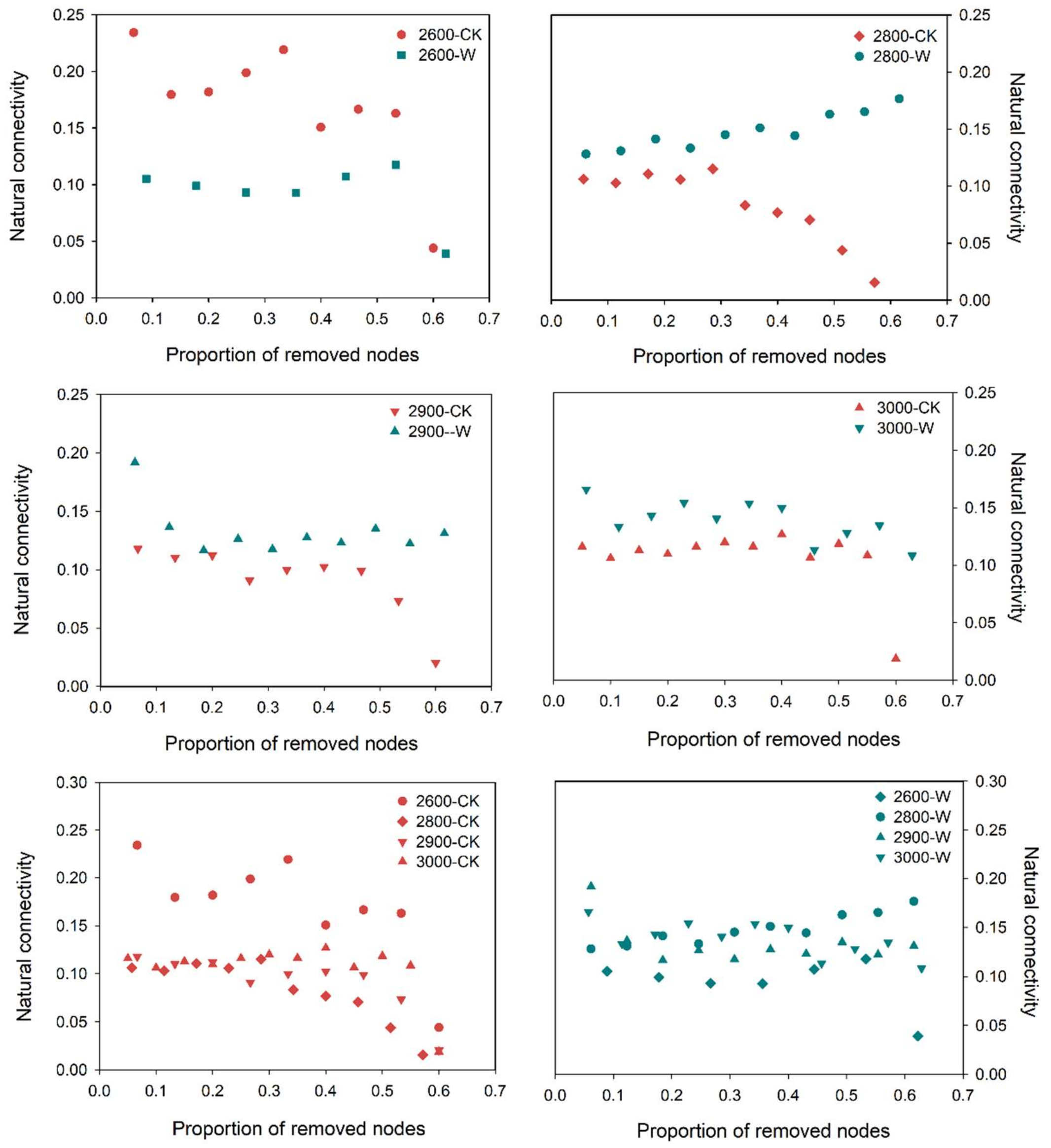

CK: Control; W: warming. Low altitude (2600 masl), medium altitude (2800 masl), medium-high altitude (2900 masl), and high altitude (3000 masl).
